# Development of online classification system for construction waste based on industrial camera and hyperspectral camera

**DOI:** 10.1371/journal.pone.0208706

**Published:** 2019-01-16

**Authors:** Wen Xiao, Jianhong Yang, Huaiying Fang, Jiangteng Zhuang, Yuedong Ku

**Affiliations:** Key Laboratory of Process Monitoring and System Optimization for Mechanical and Electrical Equipment (Huaqiao University), Fujian Province University, Huaqiao University, Xiamen, Fujian Province, China; Newcastle University, UNITED KINGDOM

## Abstract

Construction waste is a serious problem that should be addressed to protect environment and save resources, some of which have a high recovery value. To efficiently recover construction waste, an online classification system is developed using an industrial near-infrared hyperspectral camera. This system uses the industrial camera to capture a region of interest and a hyperspectral camera to obtain the spectral information about objects corresponding to the region of interest. The spectral information is then used to build classification models based on extreme learning machine and resemblance discriminant analysis. To further improve this system, an online particle swarm optimization extreme learning machine is developed. The results indicate that if a near-infrared hyperspectral camera is used in conjunction with an industrial camera, construction waste can be efficiently classified. Therefore, extreme learning machine and resemblance discriminant analysis can be used to classify construction waste. Particle swarm optimization can be used to further enhance the proposed system.

## Introduction

Recent technical developments have been focusing on protecting the environment and saving resources. Several studies have discussed the hazardous effects of accumulating construction waste [1]. Construction waste includes materials such as plastics, concrete, wood, and bricks that can degrade the environment; however, these materials can be extremely valuable when recycled or repurposed because they are manufactured via complicated procedures. They are composed of limited nonrenewable resources. Therefore, complicated construction waste must be efficiently separated.

Some studies have used near-infrared hyperspectral camera to classify different objects [2,3]. This camera can capture the internal composition of objects by exploiting the DN values in different wavebands. The name hyper emanates from the camera’s ability to capture hundreds of wavebands with high resolution. By analyzing these DN values, objects can be classified based on their composition. The generated hyperspectral data are processed and displayed as a curve with hundreds of DN values. In practical scenarios, hyperspectral data have numerous dimensions that compromise the calculation efficiency. Therefore, some studies have investigated different ways of extracting feature-related wavebands [4–6]. The most recent ways involving classification using hyperspectral data include extreme learning machine (ELM) [7–9], partial least squares discriminant analysis (PLS-DA) [10,11], and various deep learning algorithms [12,13]. Several other studies have focused on further improving the classification accuracy [14–16].

Thus, the technology can efficiently classify objects using hyperspectral data. However, some problems pertaining to the usage of this technology still need to be addressed. The hyperspectral camera is presently used to acquire spectral data. In most situations, this classification is executed offline via linear scanning and imaging. This imaging is time-consuming, and the amount of generated spectral data will increase over time that can be up to several decades. Furthermore, the need for capturing hyperspectral data hundreds of times makes it difficult to achieve online classification. This study proposes a method that can efficiently achieve online classification.

## Materials and methods

**Fig 1:**
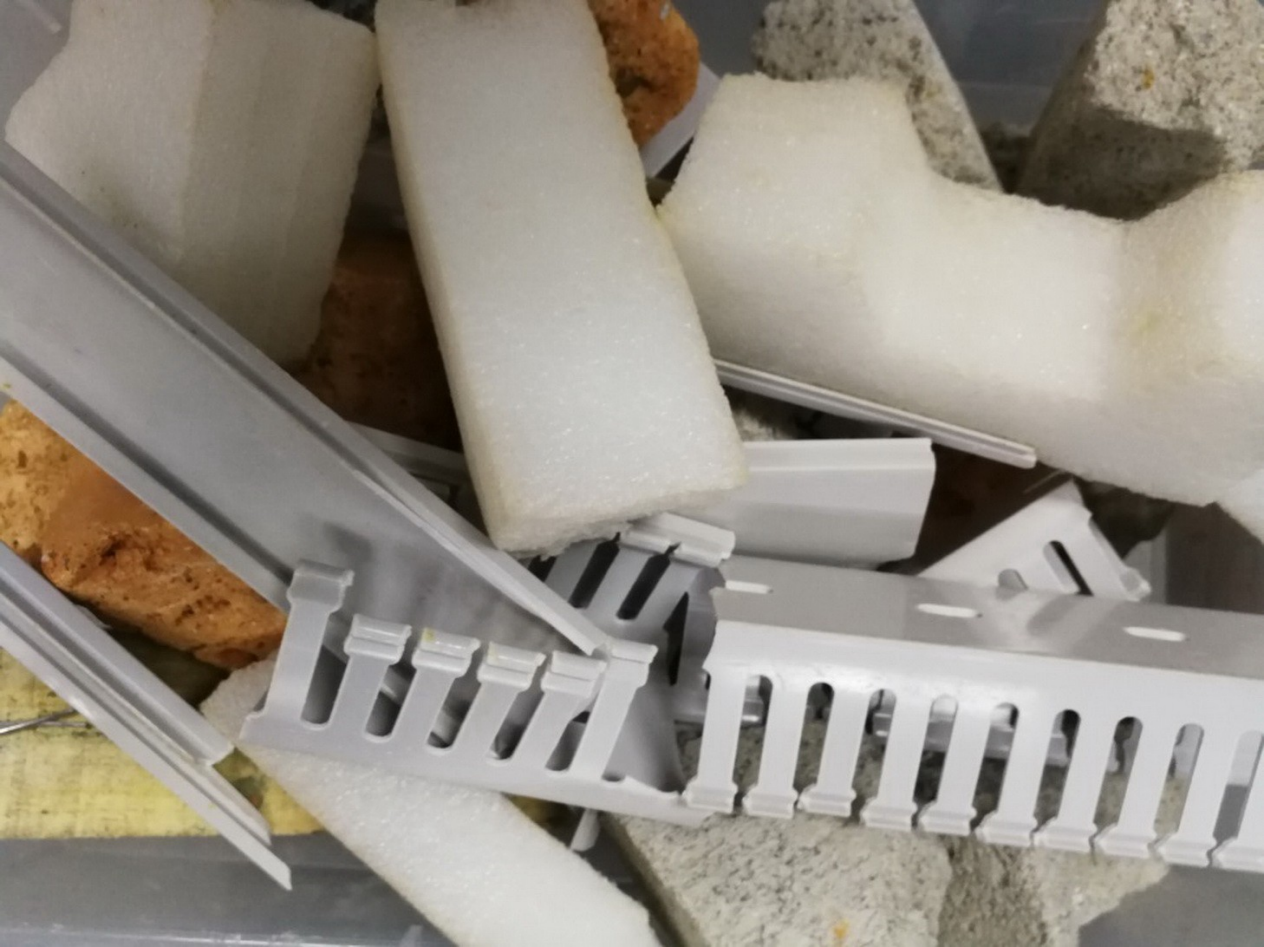
Some construction waste samples.

This study mainly aimed to study the classification of construction waste. Some common construction waste samples were obtained in the form of plastics, bricks, concretes, and woods; 80 pieces of each material were obtained. Among these, 50 pieces were used as the training set. The remaining 30 were used as the testing set. Some of these samples are shown in Fig 1. Among them, the foam comes from various packaging boxes, the plastic comes from the electric control cabinet trunking and the plastic pipe, the brick is ordinary red brick, the concrete comes from the abandoned building, the wood is the wooden box board and the dilapidated table and chair.

### A. Capturing system

**Fig 2:**
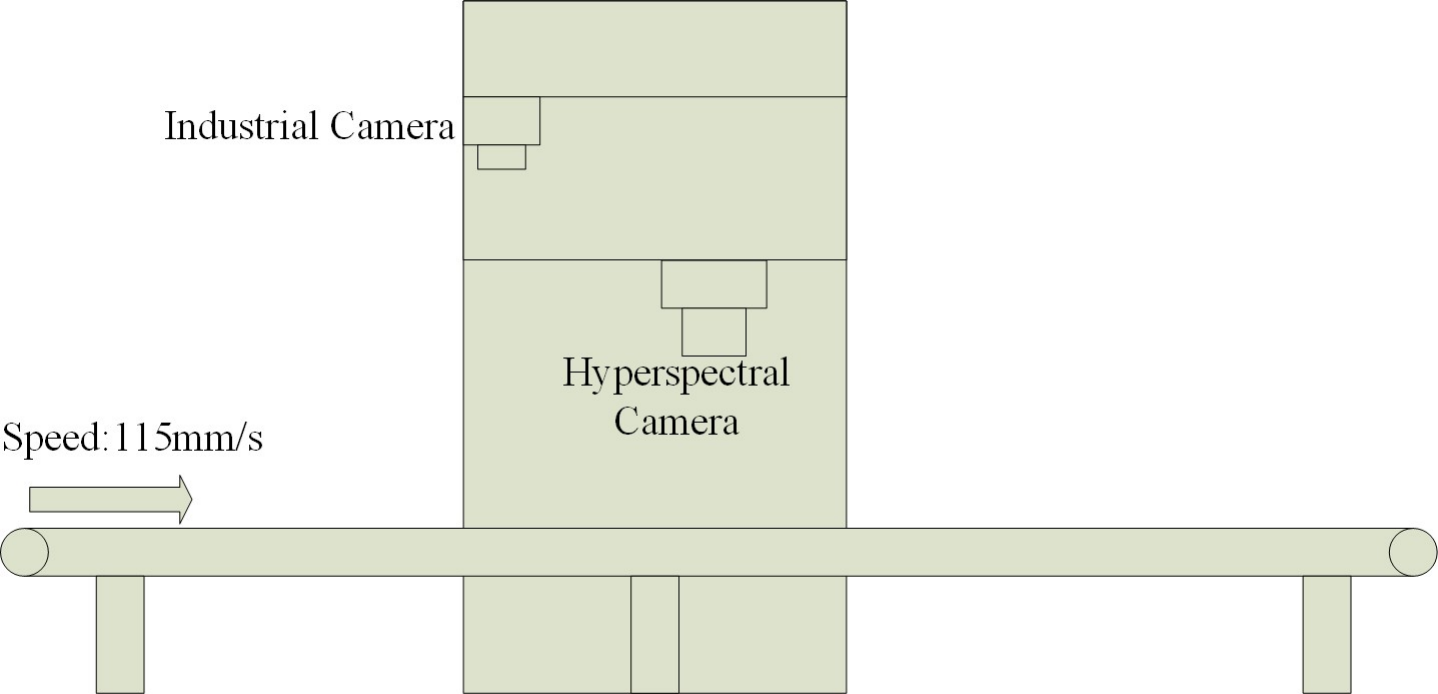
System’s structure.

**Fig 3:**
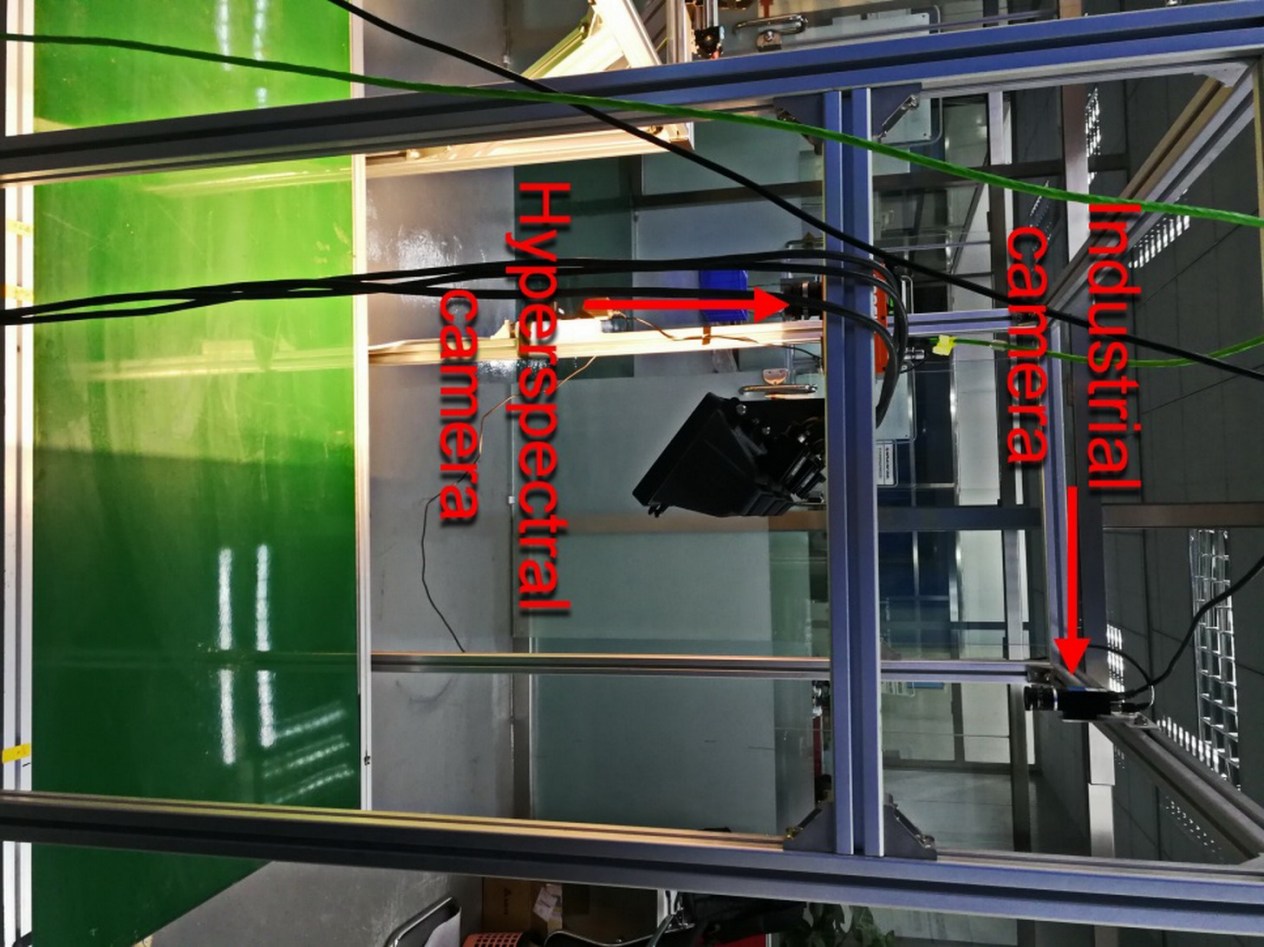
Real system.

The system’s structure as shown in Fig 2. The capturing system mainly has two components. The front is a 2D industrial camera with 640*480 pixels, and the rear is a near-infrared (NIR) hyperspectral camera with 640 spatial pixels and 224 spectral pixels. The hyperspectral camera has a wavelength range of 900–1700 nm. The system runs in the order: the objects are transported by belt to the industrial camera work area, and the industrial camera collects 2D pictures. The 2D pictures can be used to obtain the outline and centroid of the objects; Centering on the centroid of the objects, take a 5 * 5 rectangular pixels range as the region of interest (ROI) for the objects. The ROI of object passes through the working area of the NIR hyperspectral camera. The hyperspectral camera collects the spectral data in the ROI and identifies the material, and the recognition result of the ROI is used as the recognition result of the object. The parameters of the system are: belt speed is 115mm/s, industrial camera frame rate is 10fps, and hyperspectral camera frame rate is 120fps. At last, Fig 3 shows the real system.

The C++ language is used to control and analyze the data of this system, thereby enabling a convenient development of follow-up processes such as the recycling sorting system.

### B. Data processing

#### (I)Preprocessing

The original data required correction by white data and black data to ensure a standard reflectance and minimize variations influenced by light source differences. The calibrated formula is as follows:

$$\frac{D-D_{B}}{D_{W}-D_{B}}$$ (1)

where *D*, *D*_*B*_, *D*_*W*_ are the DNs of the original data, pure black pixels, and standard white board, respectively.

*D*_*B*_ is obtained by capturing an image by covering the lens cap, and *D*_*W*_ is obtained by capturing the image of a standard white board. The standard white board is made by poly tetra fluoro ethylene (PTFE). This approach allows eliminating the bias that could be introduced by light intensity and other environmental factors.

The spectral curve of any pixel is an independent one-dimensional curve signal. Due to external environmental factors and the internal components of the camera, the data collected by the camera has more or less noise, which will have some influence on subsequent feature wavebands selection and material identification. Therefore, the collected reflectance data needs to be denoised. Using the Savitsky–Golay (SG) algorithm to denoise the spectral curve can achieve good results and it is easier to implement. This article uses a 7-point smooth fit with a fitting formula of:

$$y_{0}=(-2(x_{-3}+x_{+3})+3(x_{-2}+x_{+2})+6(x_{-1}{+x}_{+1})+{7x}_{0})/21$$ (2)

where *x* is the input data, *y* is the smoothed output data, the subscript "0 " indicates the position of the current point, and the "+*n* " subscript indicates the point n to the right of the current point, otherwise the "-*n*" subscript indicates the point n to the left of the current point. Eq (2) only shows the formula of the total length of the data minus 6 points and the calculation method of the edge point can refer to [17].

#### (II)Wavebands selection

Spectral data have numerous dimensions, which exhibit strong relations with approaching bands. These redundancies complicate the data analyses and make the process time-consuming, thereby making spectral data unsuitable for online analysis. This problem can be resolved using feature vector to replace all data; however, the most representative feature vector must be determined. In this study, two typical algorithms are used to extract feature bands. After extensive optimization, a method to execute this procedure is derived.

The principle component analysis (PCA) [3] enables extracting spectral feature wavebands and yields good result. The main objective of this algorithm is to reduce the dimensions of the data. According to the components of every score, the original data can be translated into fewer dimensions. During this procedure, a matrix coefficient, whose crests and troughs can to some extent represent the character of original data, is loaded. The signatures of these crests and troughs in all spectral data are known as features.

The successive projections algorithm (SPA) [6] separates original matrix to several small matrices. Then, each vector belonging to a matrix is used to project others vectors. Each small matrix will contain unrelated vectors, whose situation at original matrix will be the features. Then, some feature wavebands will be obtained but not the least. The best result from all of the acquired results is determined via testing accuracy.

### C. Experimental program

**Fig 4:**
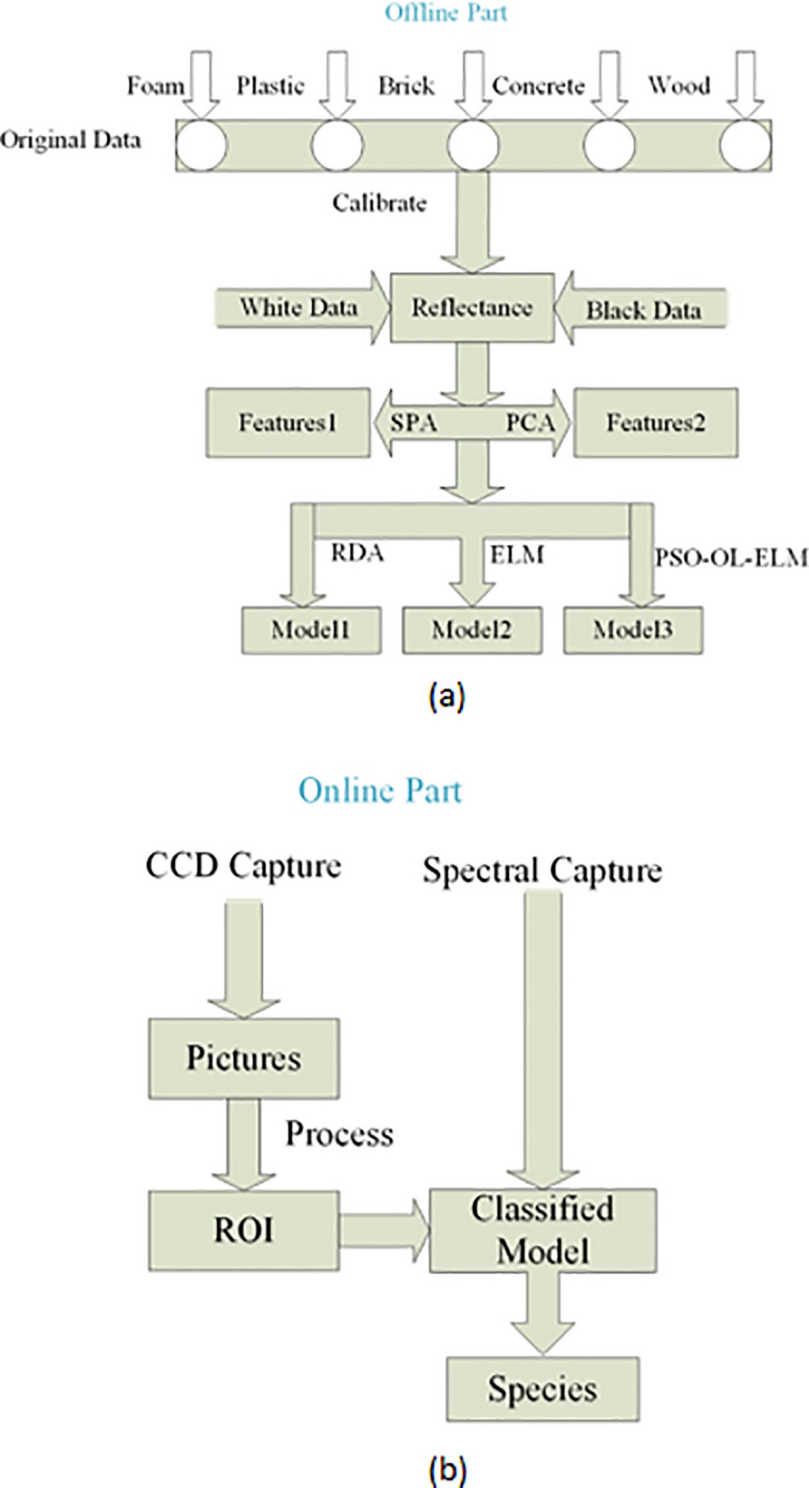
**The procedure of system**: (a) Offline part, (b) Online part.

As shown in Fig 4, this classification system comprises offline and online parts. The main objective of the offline part is to build the classification model. In this study, SPA and PCA are used to extract features; then, the classified model is developed using resemblance discriminate analysis (RDA), extream learning machine (ELM) and particle swarm optimization and online learning extream learning machine (PSO–OL–ELM), respectively. The online part uses the industrial camera to capture ROI, the hyperspectral camera to capture the spectral data, and finally the classification model to classify objects into different species.

The accuracy of this system is mostly dependent on the classification model. Therefore, the results from three different models are compared and the best result is adopted.

#### (I) Classification based on ELM

The ELM is a simple, single hidden-layer forward neural network that was developed by professor Hunang [18]. The ELM uses the Moore–Penrose to calculate the weight of output matrices. This algorithm can be quickly trained without compromising the accuracy of the results.

**Fig 5:**
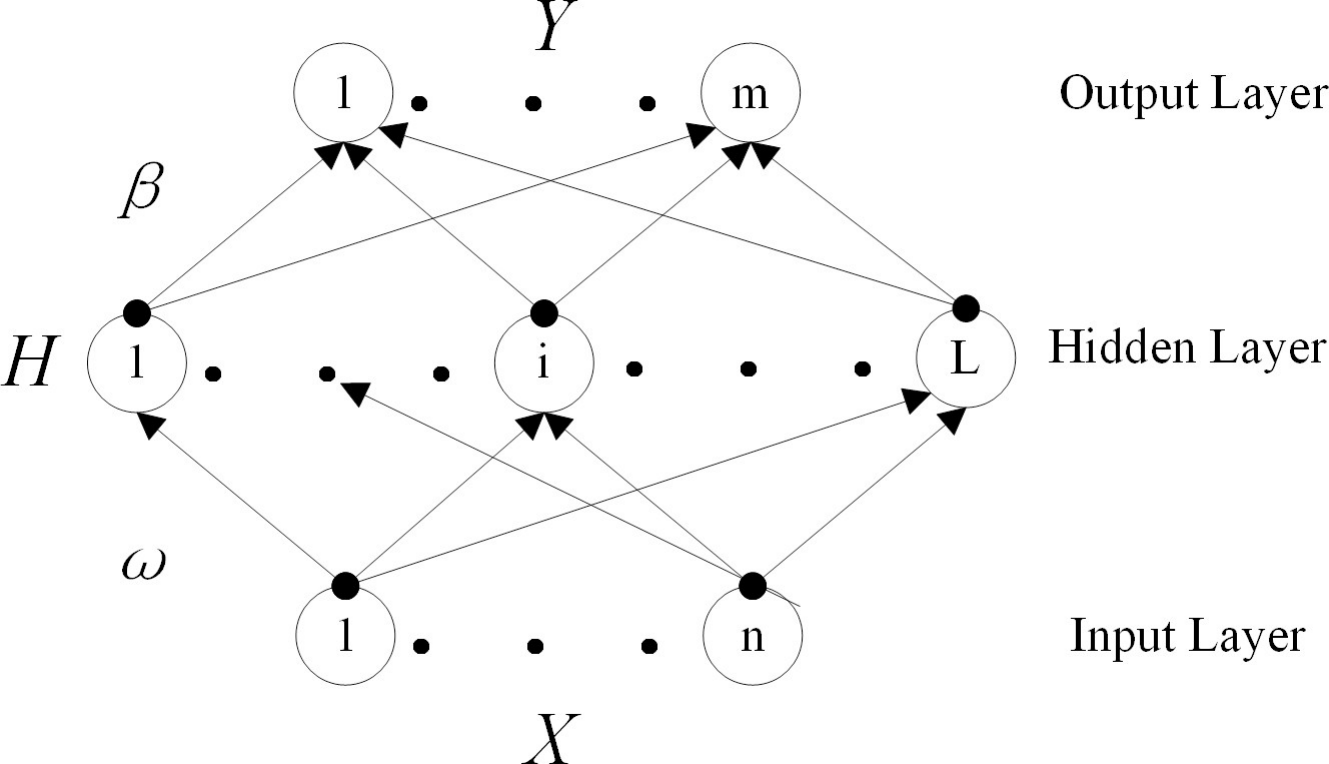
Structure of ELM.

The structure of ELM as shown in Fig 5. The weights of input layer with hidden layer *ω*_*n*×*L*_ can be written as follows:

$$\omega =\begin{array}{cccc} \omega_{11} & \omega_{12} & \ldots & \omega_{1L}\\ \omega_{21} & \omega_{22} & \ldots & \omega_{2L}\\ \ldots & \ldots & \ldots & \ldots \\ \omega_{n1} & \omega_{n2} & \ldots & \omega_{nL} \end{array}$$ (3)

The weights of hidden layer with output layer *β*_*L*×*m*_ can be written as follows:

$$\beta =\begin{array}{cccc} \beta_{11} & \beta_{12} & \ldots & \beta_{1m}\\ \beta_{21} & \beta_{22} & \ldots & \beta_{2m}\\ \ldots & \ldots & \ldots & \ldots \\ \beta_{L1} & \beta_{L2} & \ldots & \beta_{Lm} \end{array}$$ (4)

The training data can be separated into two matrices *X*_*k*×*n*_ and *Y*_*k*×*m*_. The *k* is the size of data, every data will have *n* feature bands. Then, *X* and *Y* can be represented as follows:

$$X=\begin{array}{cccc} x_{11} & x_{12} & \ldots & x_{1n}\\ x_{21} & x_{22} & \ldots & x_{2n}\\ \ldots & \ldots & \ldots & \ldots \\ x_{k1} & x_{k2} & \ldots & x_{kn} \end{array}$$ (5)

$$Y=\begin{array}{cccc} y_{11} & y_{12} & \ldots & y_{1m}\\ y_{21} & y_{22} & \ldots & y_{2m}\\ \ldots & \ldots & \ldots & \ldots \\ y_{k1} & y_{k2} & \ldots & y_{\mathrm{km}} \end{array}$$ (6)

Substitute *n* with the number of species. *X* is the feature, and *Y* is the label comprising 1 and 0. There were *m* species, which species belong to make that number be 1, others be 0. To complete this function, assuming that *B*_1×*L*_ is the basis of the hidden layer, we can show that

$$B=[b_{1},b_{2}\ldots b_{L}]$$ (7)

A matrix *H* can be assumed to represent the output of hidden layer; then, *H* can be obtained according to the following function:

H=G(Xω+B) (8)

where *G* is the activating function represented by the sigmoid as follows:

$$g(x)=\frac{1}{1+e^{-x}}$$ (9)

For the most essential process, the Moore–Penrose of *H*, which is called *H*^+^, must be calculated as follows:

$$O=H\beta \Rightarrow \beta =H^{+}O$$ (10)

And then $H^{+}=(H^{T}H+\frac{I}{C}{)}^{-1}H^{T}$, *I* is identity matrix and *C* is penalty factor. A suitable *C* can avoid overfitting.

*β* can be calculated if *Y* is used instead of *O* when training. After completing the training, the computation from the beginning to the end of this network can be used to obtain y, which represents the species of this new data *x*.

y=G(xW+B)β (11)

standardize y belong 0 to 1, which represent how similar was it to the corresponding species.

#### (II) Classification based on resemblance discriminate analysis (RDA)

Every data of one object are represented as a vector with *n* dimensions (where *n* is the number of feature bands). To classify objects into species, the corresponding vectors must be classified using the Euclidean distance in combination with other methods. Herein, the Euclidean distance and the subjoining angle are used to establish the difference between species.

$$d=\sqrt{(v_{1}-v_{2})(v_{1}-v_{2}{)}^{T}}$$ (12)

where *d* is the Euclidean distance between *v*_1_,*v*_2_ and *v*_1_,*v*_2_ are vectors with one row and n columns.

By referencing the formulation of two dimensions and three dimensions, the angle of two vectors with *n* dimensions can be calculated using the following function:

$$\theta =\mathrm{arc}\cos\frac{v_{1}v_{2}}{\left|v_{1}v_{2}\right|}$$ (13)

We considered every species’ center vectors as the corresponding average vectors. Then we determined the largest Euclidean distance in the training data as the species’ radius. The Euclidean distances between new data, and every species’ center can be calculated for comparison with the corresponding species’ radium. If the minimum distance between that species was smaller than homologous radius, then the data can be attributed to that species. If the minimum and second minimum distance were similar and both smaller than homologous radius, then the species’ center would need to be calculated. Lastly, we assign class to respective species using the smallest two *θ* values.

In the formula (5), *X* could be separated to *m* (the number of species) matrix *X*_1_,*X*_2_…*X*_*m*_ according to species.

$$X_{m^{'}}=\begin{array}{cccc} x_{11} & x_{12} & \ldots & x_{1n}\\ x_{21} & x_{22} & \ldots & x_{2n}\\ \ldots & \ldots & \ldots & \ldots \\ x_{j1} & x_{j2} & \ldots & x_{jn} \end{array}$$ (14)

where *m*' is the range from 1 to *m*. The center of species could be calculated using the following function:

$$C_{m^{'}i}=\frac{1}{j}\sum_{k=1}^{j}x_{\mathrm{ki}}$$ (15)

where *i* is the range from 1 to *n*.

According to formulas (12) and (13), the distance and angle can be obtained. As described above, its species can be determined.

#### (III) Improving by particle swarm optimization(PSO) and online learning(OL)

The particle swarm optimization algorithm is used to improve accuracy of classification. Online learning is simultaneously used to extend the training set. These techniques enhanced the efficiency of ELM; however, the ELM algorithm does not yield enough accurate results. For instance, this algorithm first needs to initialize *W* (weight between input and hidden layers) and *B* (bias of layer’s neurons), both of which are random. As a result, training will yield different results each time. To fix this problem, PSO algorithm is used to optimize *W* and *B* as follows:

Step 1: The size of population *N* is determined. Then, the population’s position *x* is initialized to include *(n+1)L* particles representing *W* and *B*, which randomly range from −1 to 1. At the same time, the velocity *v* of every particle is initialized randomly between their limits. On average, the velocity’s limits are between 0.25 and 0.5 position’s limits. High velocity would make the position deviate from the best result, whereas low velocity would make the position fall into the local optimum.
Step 2: The fitness function is derived as follows:

$$f(X)=\frac{1}{k}\sum_{i=1}^{k}((O_{rowi}-Y_{rowi})*(O_{rowi}-Y_{rowi}{)}^{T}{)}^{\frac{1}{2}}$$ (16)

where *k* is the size of training set, *O* is the real output, *Y* is the label of training set.

- Step 3: Each particle’s *j* fitness function is determined, and the smallest function *pbest*_*j*_ is identified. Each population’s *pbest*_*j*_ is further determined, and the smallest function *Gbest*_*i*_ is determined.
- Step 4: Each population’s particle position and velocity is updated according to the following functions (16):

$$v_{\mathrm{ij}(t+1)}=v_{\mathrm{ij}(t)}+c_{1}r_{1}(pbest_{i(t)}‑x_{\mathrm{ij}(t)})+c_{2}r_{2}(Gbest_{i(t)}‑x_{\mathrm{ij}(t)})$$ (17)

$$x_{i_{j(t+1)}}=\arg\;\min(Gbest_{ij(t)})$$ (18)

among these functions, *c*_1_ and *c*_2_ denote the studying rates, whereas *r*_1_ and *r*_2_ denote random from 0 to 1.

- Step 5: The number of iterations or its fitness is determined. If the results are satisfactory, the procedure is assumed to be completed; however, if the result is not satisfactory, step 2 of the procedure must be reiterated.

In addition, the size of the training set affects the accuracy. When new data has a similarity threshold of 70%, it will be incorporated as a part of the training data because it enhances the accuracy of the testing procedure. As shown in Table 1, 117 ms are required in 250 training datasets. The training time will increase with the increasing amount of training data. By reducing the time required for online learning by milliseconds, the model’s efficiency can be improved, particularly when more training data are involved. Therefore, the turnaround time will continuously improve.

**Table 1:** Result of ELM.

	Foam	Plastic	Brick	Concrete	Wood
Training time	117 ms				
Training set/piece	50	50	50	50	50
Testing set/piece	30	30	30	30	30
SPA result/piece	29	30	28	28	30
PCA result/piece	28	30	30	29	30
Overall accuracy	SPA: 96.667%		Overall accuracy	PCA: 98%	

To mitigate this issue, a temper matrix *P* [7] is assumed. Here, the subscript 0 represents old data and the subscript 1 represents new data.

$$P_{0}=(H_{0}^{T}H_{0}+\frac{I}{C}{)}^{‑1}$$ (19)

$$P_{1}=(P_{0}^{‑1}+H_{1}^{T}H_{1}{)}^{‑1}$$ (20)

$$\beta_{1}=\beta_{0}+P_{1}H_{1}^{T}(Y_{1}-H_{1}\beta_{0})$$ (21)

where *H* is same to function (8), the *β* is same to function (4), *I* is Identity matrix and *C* is a penalty factor. A suitable *C* can avoid overfitting. At last, using *β*_1_ to instead of the *β* in function (10) and then the new classifier can be obtained.

## Result and discussion

Owing to considerable noise, the data close to 900 or 1700 nm are excluded in the analysis. These five species are distinguished according to their spectral data. However, some species could not be classified because they resembled wood, foam, brick, and some species such as plastic and concrete.

**Fig 6:**
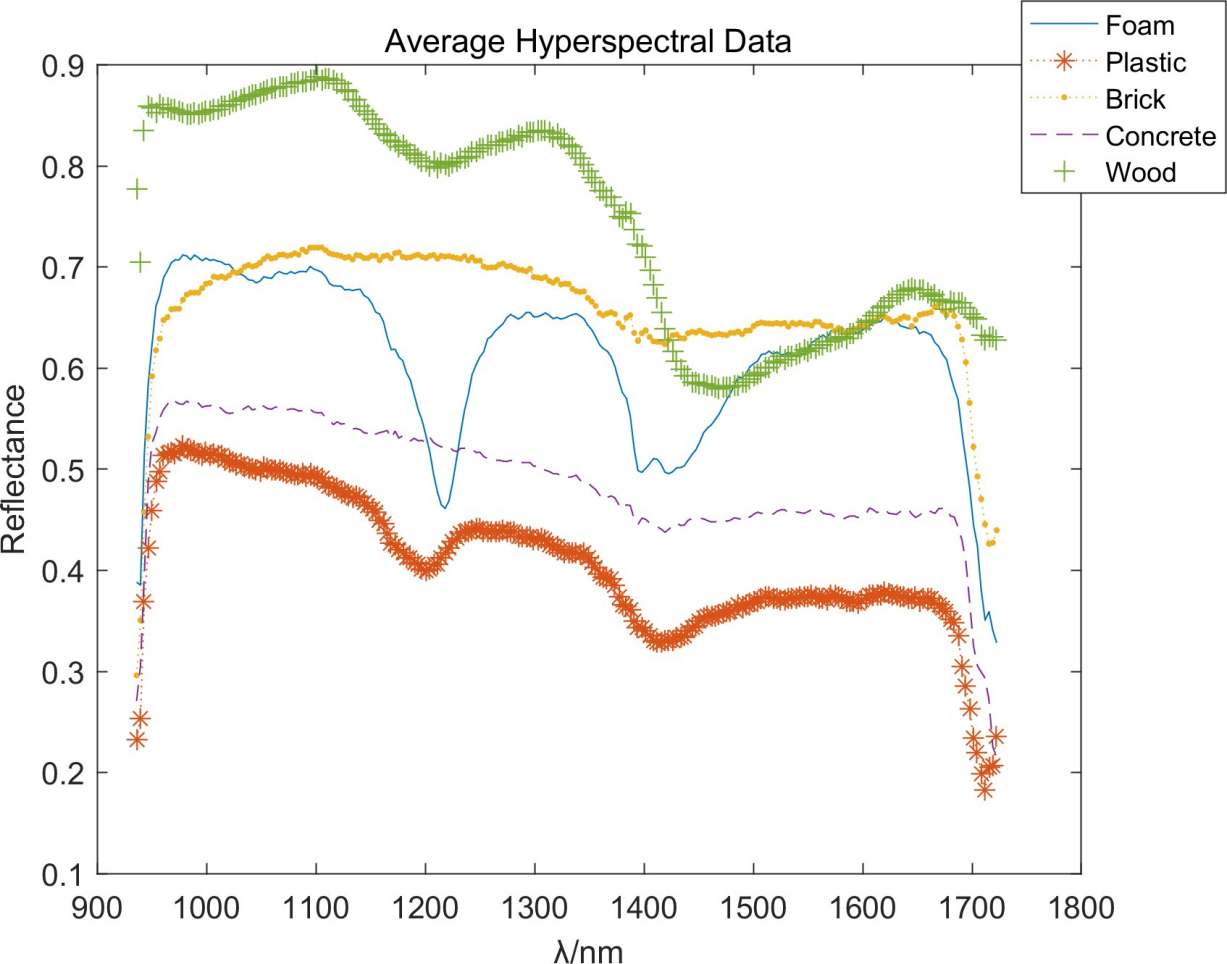
The original average reflectance curve.

The average reflectance of five materials are is showed in Fig 6. Foam, plastic, wood are organic, with obvious absorption peaks, expressed as trough characteristics on the curve, while brick and concrete are inorganic, without obvious absorption peak characteristics. The edge points of various materials are similar in some degree. The five types of materials can be clearly distinguished around 1200 and 1400 nm.

### A. Selection of feature bands

**Fig 7:**
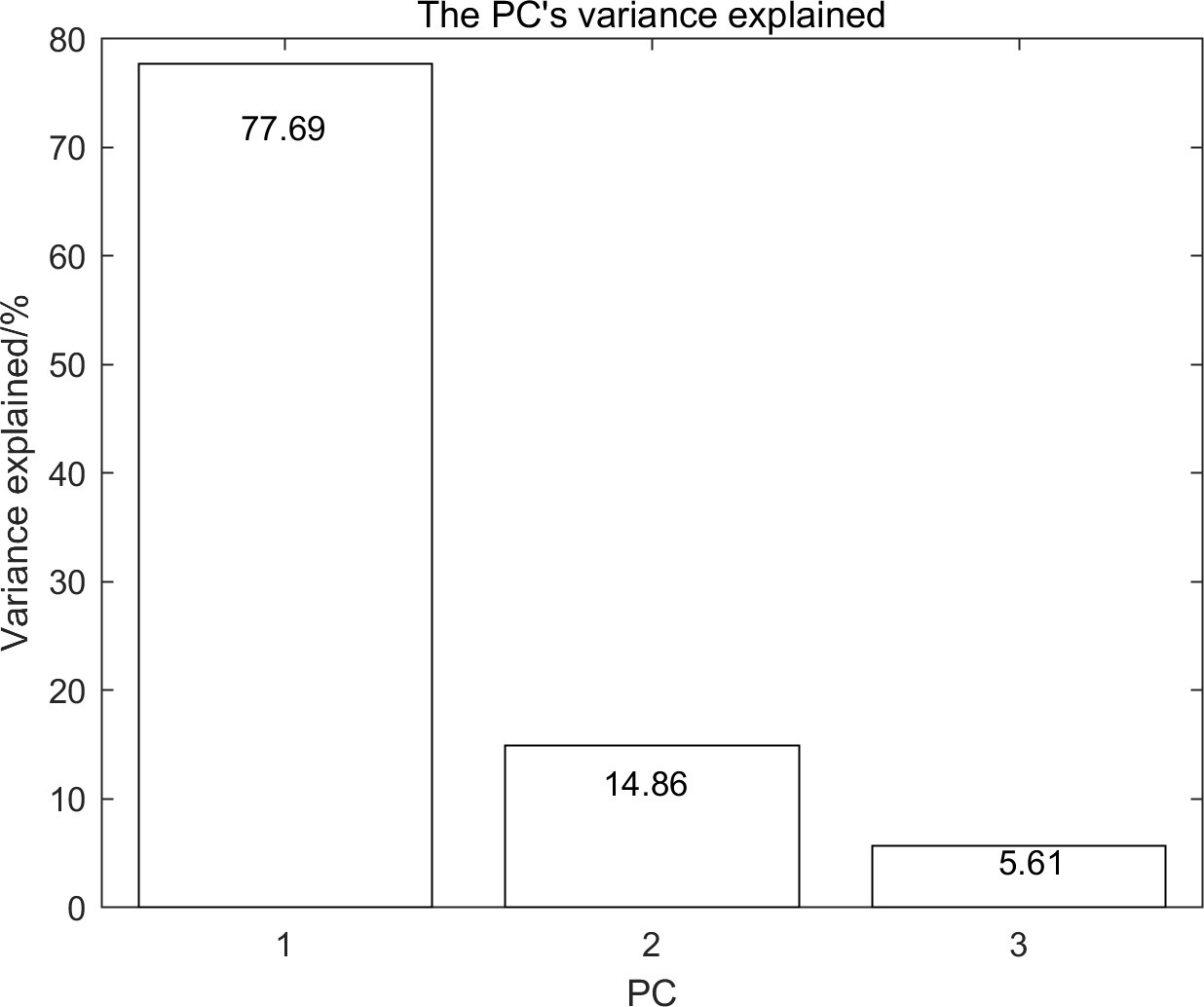
The PC’s variance explained.

The three principal components can provide 98.16% of the information about the original data. Specifically, PC1 (77.69%), PC2 (14.86%), and PC3 (5.61%), which can show original features in some degree. Therefore, these components can be used to obtain feature wavebands reliably. The 3 PC’s variance explained is showed in Fig 7.

**Fig 8:**
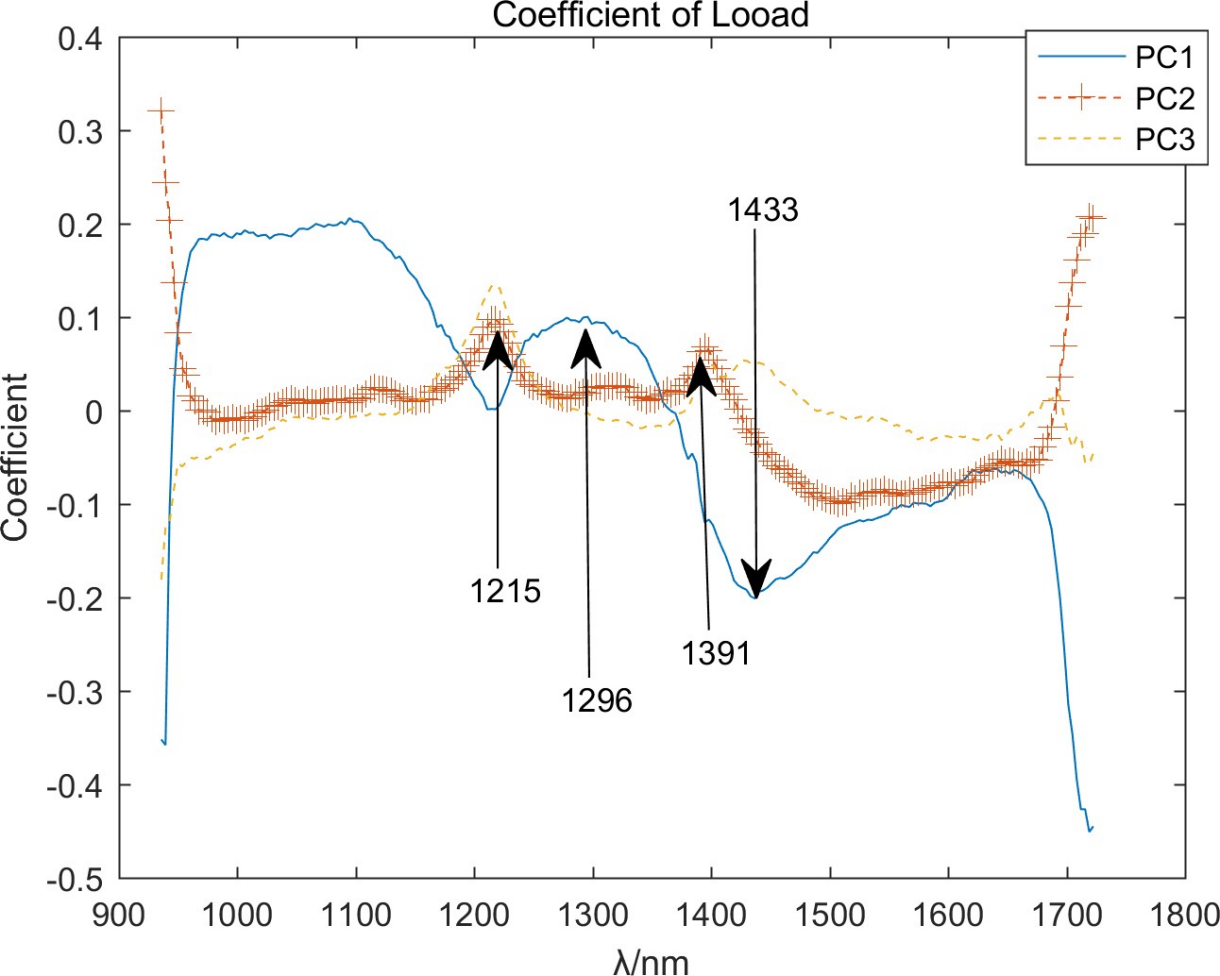
Coefficients of load.

**Fig 9:**
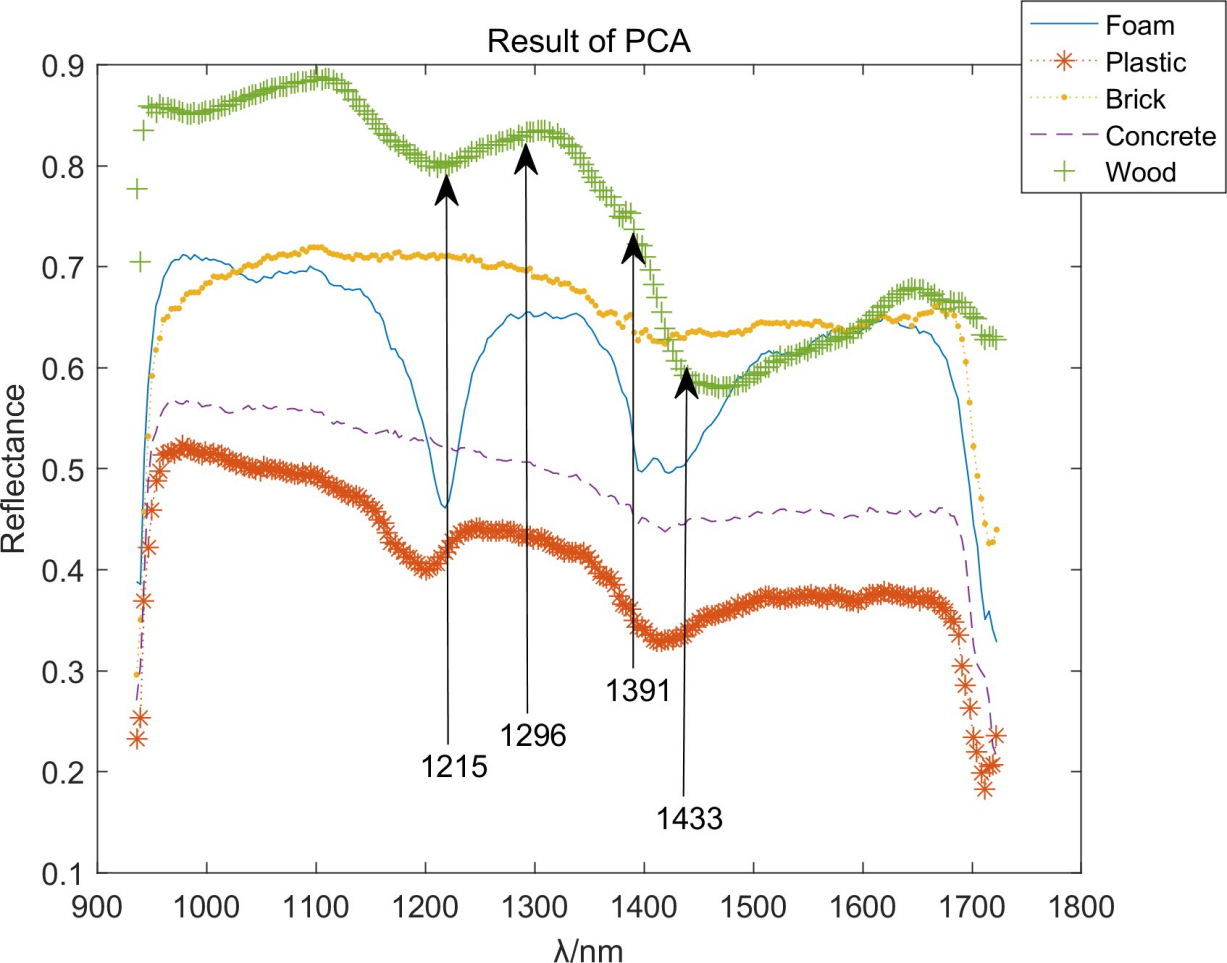
Feature bands with PCA.

The crests and troughs of the curve of load coefficient can express the difference of original curve [3]. According to the Fig 8, the features of PCA can be got, and this result can be verified using the observed feature wavebands at 1215, 1296, 1391, and 1433 nm. Therefore, this system is dependable to some extent. The feature bands with PCA are showed in Fig 9.

**Fig 10:**
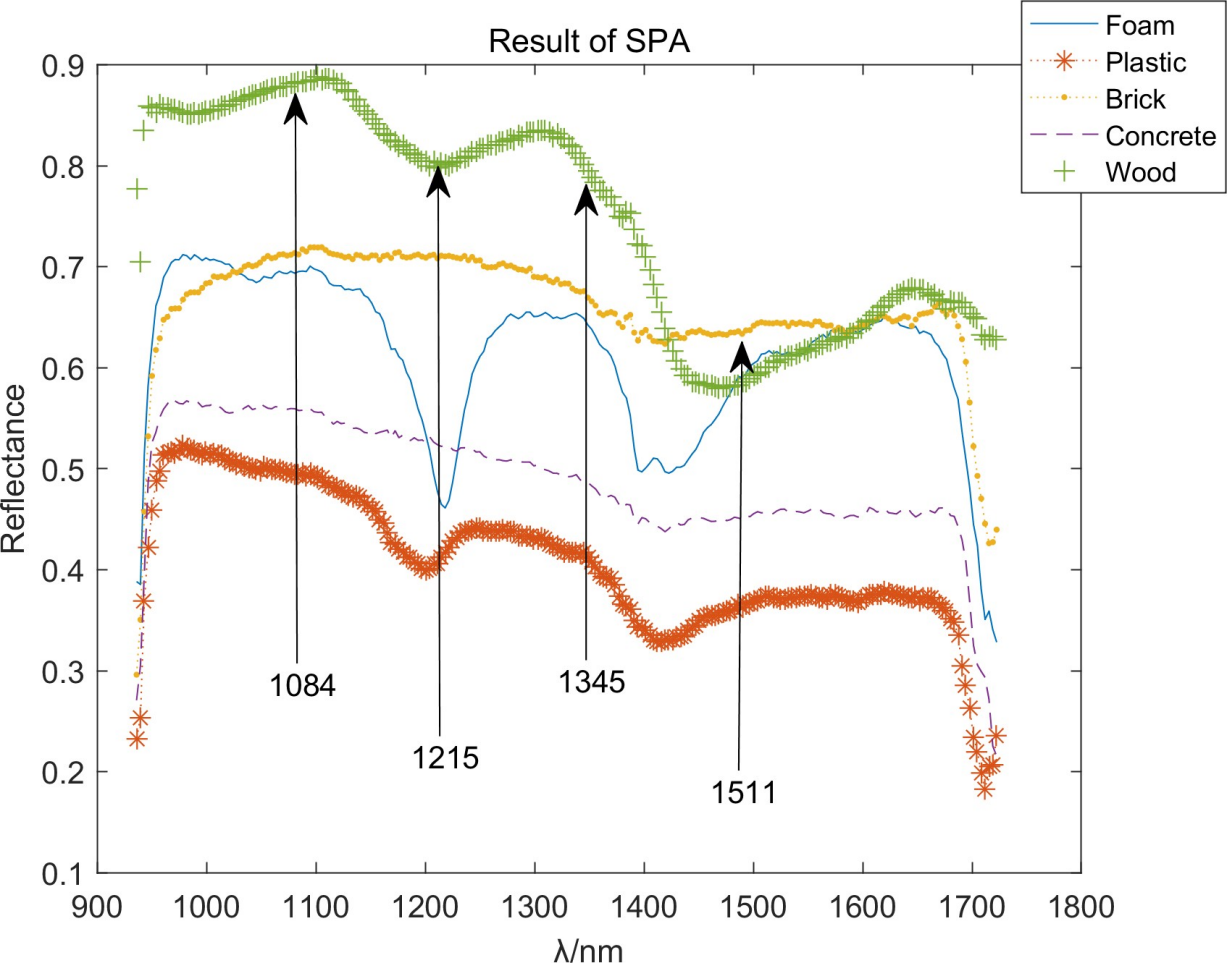
Feature bands with SPA.

The successive project algorithm separated the original matrix to the *N* subset, where *N* is the required number of feature bands. In this study, the original matrix comprised 5 rows that represented 5 species and 224 columns that represented 224 bands. We excluded the first 12 bands and last 12 bands because of the noise. The resulting matrix comprised 5 rows and 200 columns. The five types of materials are not difficult to distinguish, and do not need too many features. Refer to the number of features extracted by PCA, and set *N* = 4. As a result, there were 4 matrices with 5 rows and 50 columns each. Each matrix can be considered as 50 vectors comprising 5 factors. Each vector in one matrix projected to its respective matrix. Therefore, the minimal correlation with the matrix’s vector could be obtained; the feature bands were 1084, 1215, 1345, and 1511, and the result is showed in Fig 10.

### B. Result of experiment

The result of ELM is showed in Table 1. The overall accuracy with SPA is 96.667% and the error mainly happened in brick and concrete. The overall accuracy with PCA is 98%, slightly higher than the SPA. Although the correct rate of bricks and concrete has been improved, at the same time, the correct rate of foam has decreased slightly. Therefore, the overall accuracy of PCA extraction is higher, but the two algorithms have their own advantages and disadvantages.

In Table 2, the RDA exhibits strong calculation efficiency and classification capability because of its simplicity and strong theoretical basis. Whether it is SPA or PCA extraction features, the overall accuracy can reach 100%. It can be seen from Fig 7 and Fig 8 that there are significant differences between categories at the extracted characteristic wavelengths. A good result can be obtained by comprehensively considering the distance and angle between the sample to be tested and the standard category. In this paper, the classification weight of the distance and angle is 1:1. In different applications, the weight of the distance and angle may need to be adjusted.

**Table 2:** Result of RDA.

	Foam	Plastic	Brick	Concrete	Wood
Training time	28 ms				
Training set/piece	50	50	50	50	50
Testing set/piece	30	30	30	30	30
SPA result/piece	30	30	30	30	30
PCA result/piece	30	30	30	30	30
Overall accuracy	SPA: 100%		Overall accuracy	PCA: 100%	

Comparing the ELM and RDA, there are some conclusions. At first, ELM is a learning algorithm that do not need display programming after build the model and RDA must follow the rules given in advance. This allows the ELM to handle complex problems and be more flexible, but requires sufficient data to adequately train its model to achieve better classification results, resulting in lower accuracy when training data is insufficient. RDA is relatively rigid, but it has greater stability when dealing with simple problems and is much more efficient than ELM. At the same time, a complete RDA classifier can be implemented by only requiring representative samples of each category. However, when the obtained representative samples are insufficiently representative, the correct rate may be low, and it is difficult to obtain good results when dealing with complicated nonlinear problems.

When comparing the PCA and SPA to extract features in the same classification algorithm, PCA is considered more reliable. Although ELM in combination with PCA can yield 98% accurate results, this result is lower than RDA.

The combination of PSO and online learning can make the ELM model efficient and reliable. In this study, the size of iteration is given as 500. The results of this new algorithm are shown in Table 3.

**Table 3:** PCA–PSO–OL–ELM.

	Foam	Plastic	Brick	Concrete	Wood
Training time	1550.83s				
Training set/piece	50	50	50	50	50
Testing set/piece	30	30	30	30	30
Accuracy/piece	29	30	30	29	30
Overall accuracy	98.667%				

Thus, it is evident from Table 3 that the overall accuracy was improved, thereby indicating that the new algorithm is efficient. However, the process was time-consuming. The testing time was relatively shorter compared to the time consumed during training when the PSO was working. However, every model requires some time to execute PSO. Therefore, the current state may not be perfect, but it works. In addition, the size of testing data was small; therefore, the result did not reveal the overall accuracy. When every object matches its respective species, all of them have more similar to corresponding species. Using the average similar of matching species to assess the consequent of this way will more persuasive. The average similarity can be calculated using the following function:

$$AS_{j}=\frac{1}{k_{j}}\sum_{i=1}^{k_{j}}Y_{ij},$$ (22)

where, *j* represents species and *k*_*j*_ represents the number of species in a testing set of size *j’*.

The subscript 1, 2, 3, 4 and 5 in Table 4 refer to Foam, Plastic, Brick, Concrete and Wood.

**Table 4:** AS of ELM and PSO-OL-ELM.

	*AS*_*1*_	*AS*_*2*_	*AS*_*3*_	*AS*_*4*_	*AS*_*5*_
ELM/%	78.61	61.99	66.16	75.31	92.94
PSO-OL-ELM/%	90.54	60.00	85.42	90.14	95.74

Comparing the *AS* between PSO-OL-ELM and ELM are is showed in Table 4. Obviously, this method has improved the classification ability of this model, and there still more potential for this classification to be further refined as time goes. The PSO work in the beginning and the OL work during all running time. When the size of notes of hidden layers is enough, the effect of PSO will be lower, vice versa. Except for plastics, the remaining *AS* of species have increased. The *AS* of foam, brick, and concrete has changed a lot, while the *AS* of plastic and wood has changed less. Combined with the results of Table 1, it is indicated that the growth rate of *AS* with high error rate is large, indicating that the recognition ability of this category is improved. However the accuracy of plastic and wood are both 100%, whose capability of optimization are lower. *As* a result, the extent of *AS* changes is small, and even over-fitting occurs. For example, plastics have a slight decrease in *AS*.

## Conclusions

An online classified system of construction waste using hyperspectral and industrial cameras was demonstrated herein. The combination of near-infrared hyperspectral and industrial cameras realized an efficient online classification of construction waste. The hyperspectral camera utilized too much time and accumulated a large amount of data during imaging, which make it is hard to realize online procession. However, using industrial camera to capture and process spectral data in the regions of interest, online classification could be achieved easily. The principal component analysis and successive projection algorithms was used to extract feature wavelengths. We then successfully used RDA and ELM to discriminate construction waste. ELM is good but in this situation, RDA can perform better result than ELM. RDA and ELM have their own advantages and disadvantages. RDA is more practical, efficient, and accurate in the case of simple and easy to divide and does not have enough training data. ELM needs sufficient training data to adapt to complex situations, and the generalization performance is stronger. The correct rate is not much lower than RDA, and there is still a big improvement after PSO and OL optimization. Which identification scheme is specifically selected can be referred to this paper and determined in conjunction with actual conditions.

## Supporting information

S1 Table Average reflectance data. (XLSX)
